# PLP2-derived peptide Rb4 triggers PARP-1-mediated necrotic death in murine melanoma cells

**DOI:** 10.1038/s41598-022-06429-8

**Published:** 2022-02-21

**Authors:** Vera S. C. Maia, Rodrigo Berzaghi, Denise C. Arruda, Fabrício C. Machado, Leticia L. Loureiro, Pollyana M. S. Melo, Alice S. Morais, Alexandre Budu, Luiz R. Travassos

**Affiliations:** 1 Recepta Biopharma, São Paulo, Brazil; 2 Experimental Oncology Unit, Department of Microbiology, Immunology and Parasitology, Federal University of São Paulo, São Paulo, Brazil; 3 Integrated Group of Biotechnology, University of Mogi das Cruzes, UMC, Mogi das Cruzes, SP Brazil; 4 Department of Biophysics, Federal University of São Paulo, São Paulo, Brazil

**Keywords:** Peptides, Melanoma

## Abstract

Malignant melanoma is the main cause of death in patients with skin cancer. Overexpression of Proteolipid protein 2 (PLP2) increased tumor metastasis and the knockdown of PLP2 inhibited the growth and metastasis of melanoma cells. In the present work, we studied the antitumor activity of peptide Rb4 derived from protein PLP2. In vitro, Rb4 induced F-actin polymerization, prevented F-actin depolymerization and increased the ER-derived cytosolic calcium. Such effects were associated with necrosis of murine melanoma B16F10-Nex2 cells and with inhibition of the viability of human cancer cell lines. Loss of plasma membrane integrity, dilation of mitochondria, cytoplasm vacuolation and absence of chromatin condensation characterized tumor cell necrosis. Cleavage of PARP-1 and inhibition of RIP1 expression were also observed. In vivo, peptide Rb4 reduced the lung metastasis of tumor cells and delayed the subcutaneous melanoma growth in a syngeneic model. Rb4 induced the expression of two DAMPs molecules, HMGB1 and calreticulin, in B16F10-Nex2. Our results suggest that peptide Rb4 acts directly on tumor cells inducing the expression of DAMPs, which trigger the immunoprotective effect in vivo against melanoma cells. We suggest that peptide Rb4 is a promising compound to be developed as an anticancer drug.

## Introduction

Peptides play an important role in cell biology and in many diseases including cancer. Peptides can be used in early diagnosis, prognostic and treatment of neoplasias^1^. The main obstacles of chemotherapy are the lack of specificity on tumor targeting, which may cause side effects and resistance to multiple drugs. Hence, a need to develop new therapeutic agents is, therefore, recognized^2,3^. In recent years, peptide-based chemotherapy has received increasing attention not only because peptides can bind to the membranes of tumor cells, but also because they have low molecular weights and good cell-tissue penetration^4^. Peptides can be used in a variety of ways, as cell reagents, ligands, vaccines, hormones, carriers of cytotoxic drugs, and radionuclides^5^. Peptides that bind to specific molecular targets on tumor cells can regulate tumor cell biosynthesis or serve as a drug delivery system. Other peptides may induce specific T cell responses targeting tumor cells^6^. Various therapeutic peptides have been selectively designed to inhibit signal transduction pathways^7^ and the cell cycle^8^, induce cell death^9^, target tumor suppressor proteins^10^ as well as block transcription factors^11^. Peptides have specificity to bind and modulate a protein interaction of interest, for example they can inhibit the interaction of two proteins^12^ or act as an antagonist binding to a specific receptor^13^.

Proteolipid protein 2 (PLP2) is a small transmembrane lipoprotein of 152 amino acids that was initially found in human colonic epithelial cells^14^. Breitwieser et al.^15^ reported that PLP2 multimerizes and functions as an ion channel due to the similarity of its hydropath profile with that of a subunit of the H+ vacuolar ATPase. This hydrophobic lipoprotein is localized to the endoplasmic reticulum and A4/PLP2 has been reported to interact with Bap31^16^. PLP2, a four-transmembrane domain protein, has contributed to the tumor formation and metastasis of murine melanoma in a syngeneic B16F10 model^17,18^. A reduced PLP2 expression led to growth inhibition of B16BL6 cells in vivo and prevented detectable metastasis from primary tumor cells by decreasing fibronectin and laminin, and by reducing the migratory ability of B16BL6 cells^18^.

In this study, we investigated the cytotoxicity of Rb4, the N-terminal PLP2 peptide, against B16F10 murine melanoma in a syngeneic model. In vitro, we demonstrate that Rb4 induces biochemical and morphological alterations characteristic of necrosis. The Rb4-induced necrosis involves alteration of F-actin dynamics, increase of cytosolic calcium from the endoplasmic reticulum (ER) and the expression of two DAMPs (damage-associated molecular patterns), HMGB1 and calreticulin. Furthermore, we report on the therapeutic effect of Rb4 in a well-established metastatic and subcutaneous murine melanoma model.

## Results

### Rb4, the N-terminal PLP2 peptide, has cytotoxic activity in human and murine tumor cell lines

**Figure 1:**
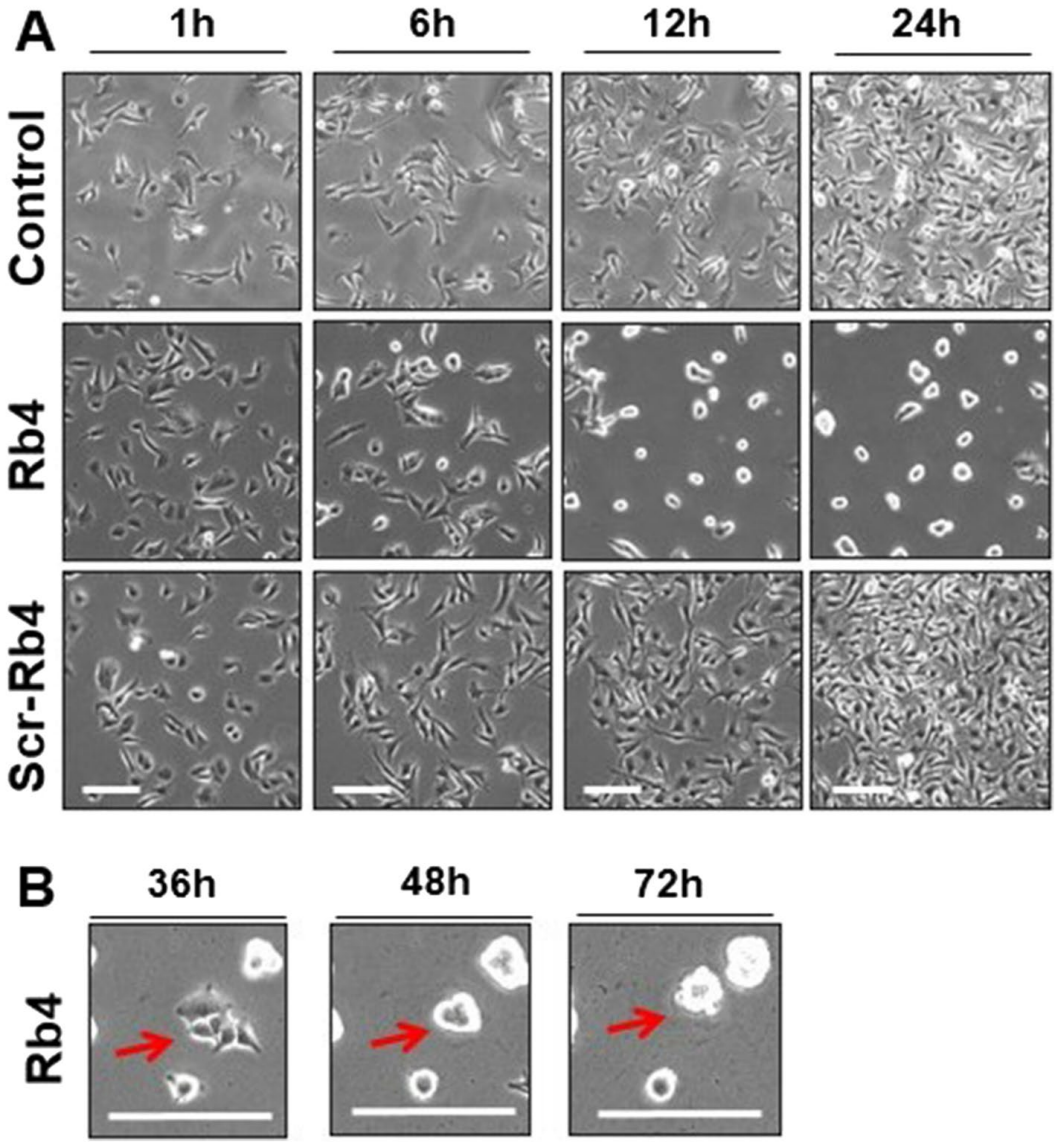
Time-lapse phase images of melanoma B16F10-Nex2 cells treated with Rb4 and Scr-Rb4. (**A**) Panels are representative fields of B16F10-Nex2 cells after control, Rb4 or Scr-Rb4 treatment for 1, 6, 12 and 24 h of incubation. Loss of morphology and no replication was observed for Rb4-treated cells. (**B**) Representative panels of B16F10-Nex2 cells treated with Rb4 for 36, 48 and 72 h showing resistant cells, highlighted in red, losing morphology and clustered after prolonged exposure to Rb4 peptide. Bar, 200 µm. See Supplementary material for full time-lapse videos.

Investigation on the cell growth and morphology was carried out in B16F10-Nex2 melanoma cells cultivated in RPMI medium with 0.15 mM Rb4 and Scr-Rb4, a scrambled Rb4 peptide, using a Nikon Biostation IMQ, an integrated cell incubator and microscopy system for long-term and multi-point live cell imaging. Pictures in Fig. 1 were extracted from time-lapse movies as shown in the Supplementary videos. Only the Rb4 sequence was able to interfere with melanoma morphology, replication and association. Rb4-treated cells did not replicate and rapidly formed clusters, absent in the control and Scr-Rb4-treated cells (Fig. 1A). Resistant Rb4 cells after 24 h of incubation also lost their natural morphology and formed clusters between 36 and 72 h of treatment (Fig. 1B).

Rb4 also inhibited other mouse and human cell lines, with different EC_50_. After 24 h under normal growth conditions (Table 1), some human cell lines, uterine cervix cancer and glioblastoma, were more sensitive to Rb4, whereas others such as breast and human melanoma, were less sensitive. Murine colon and pancreatic tumor cell lines did not respond to Rb4. Mouse embryonic fibroblasts (MEF) were resistant to Rb4.

**Table 1:** Antiproliferative activity of Rb4 peptide on tumor cell lines and non-tumor forming cell lines, ± standard deviation (s.d.).

Cell lineage	EC_50_ (mol/L × 10^–3^)^a^
B16F10-Nex2	0.263 ± 0.585
HeLa	0.525 ± 0.160
U87	0.427 ± 0.053
A2058	0.744 ± 0.208
HCT-8	> 1
MCF7	0.724 ± 0.340
PANC02	> 1
CT26	> 1
MEF	> 1

^a^EC_50_ is the concentration that decreases viability by 50% in a dose-dependent survival curve.

### Rb4 exerts effective antimelanoma activity in vivo dependent on the immune system

**Figure 2:**
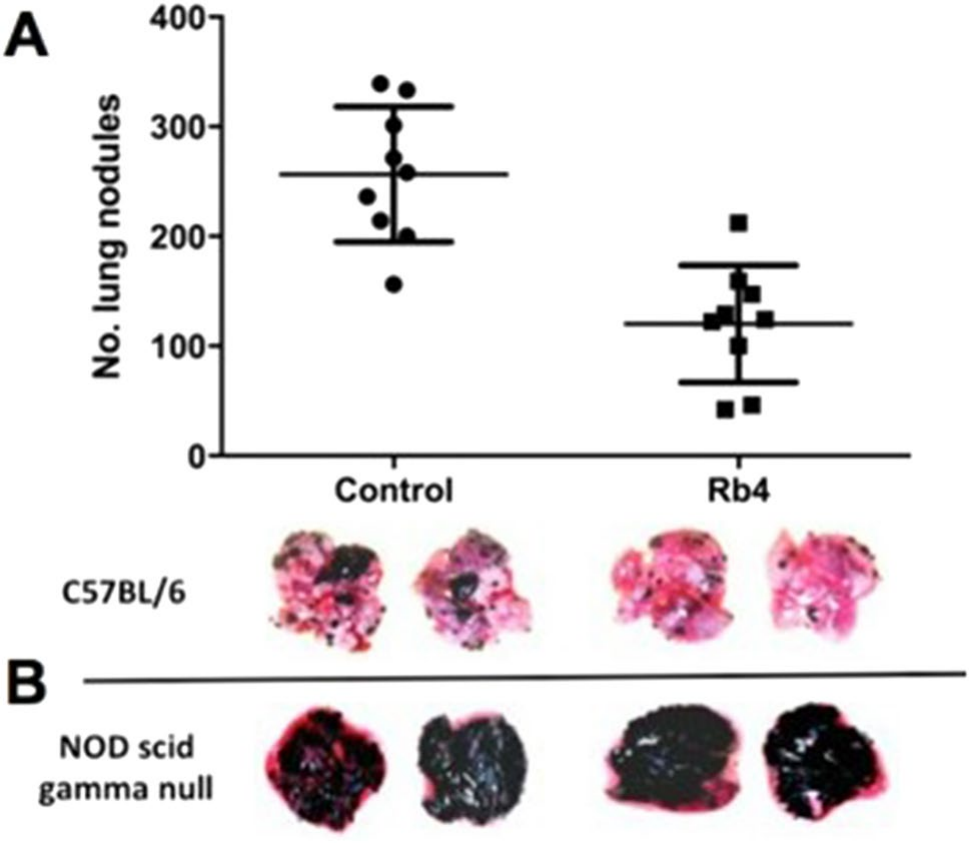
Effect of Rb4 on the protection against melanoma lung colonization in C57Bl/6 mice. (**A**) The graphic represents one of three independent experiments (n = 10). Two-tailed t-test (p < 0.01). Control: mice inoculated with peptide vehicle. Lack of protective effect of Rb4 against melanoma lung colonization in Nod/Scid IL-2R-gamma null (NSG) mice (n = 6). Control: NSG mice inoculated with vehicle.

**Figure 3:**
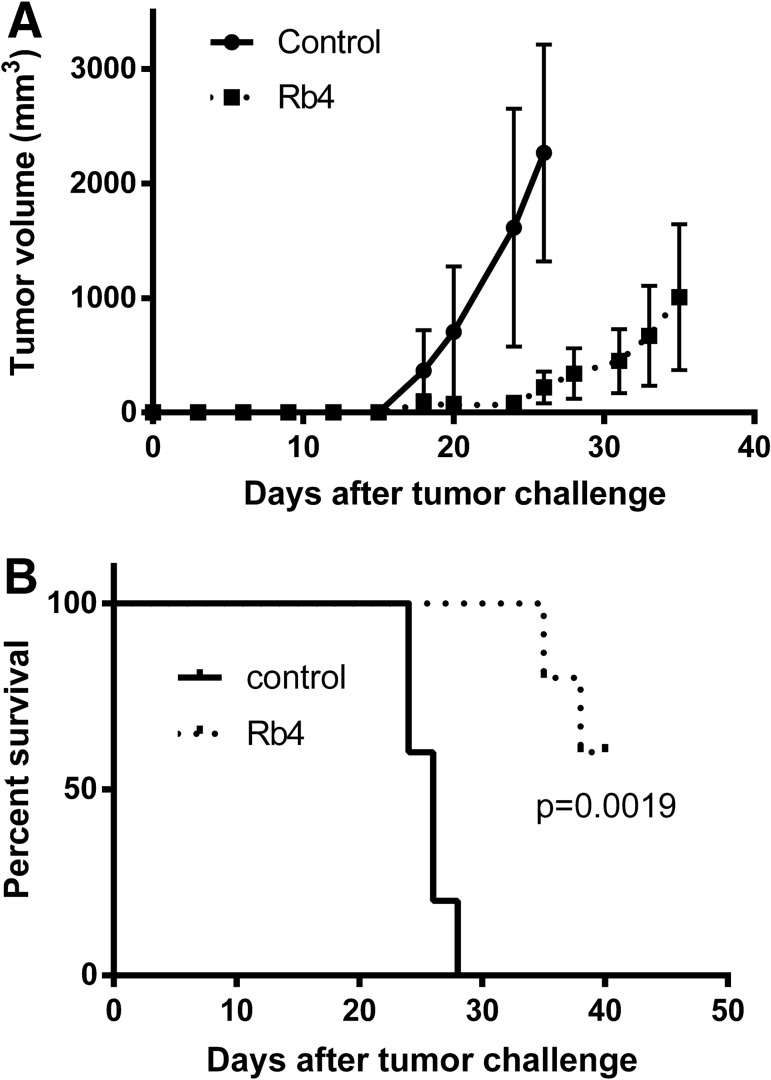
Effect of Rb4 on the protection against subcutaneous melanoma in C57Bl/6 mice. (**A**) Tumor growth curve and (**B**) Percent survival. Control: mice inoculated with vehicle. The graphic represents one of three independent experiments (n = 5). The long-term survival was recorded according to Kaplan–Meier analysis and log-rank (Mantel–Cox) test. Error bars represent the mean ± SD.

The Rb4 peptide significantly (p < 0.01) reduced the number of lung metastatic nodules in the syngeneic B16F10-Nex2 melanoma model (Fig. 2A). Melanoma cells were injected intravenously in C57Bl/6 mice and treatment consisted of 5 i.p. injections of 300 μg of Rb4 peptide or vehicle (10% water and 90% PBS), in alternate days, starting the day after tumor cell challenge. The Rb4 protective activity was also evaluated using subcutaneous B16F10-Nex2 melanoma grafted syngeneic mice. The peptide was injected i.p., with 5 doses of 300 μg/animal in alternate days, which delayed the tumor growth up to 40 days (Fig. 3A). Moreover, the survival rate of mice treated with Rb4 was significantly (p = 0.0019) greater than that of vehicle control groups (Fig. 3B), increasing group survival more than 25% and up to 10 days. In addition, this protective activity was absent in the immunocompromised NOD/SCID (Fig. 2B). Like treatment in the immunocompetent C57Bl/6 mice, immunocompromised NOD/SCID mice were inoculated intravenously with melanoma cells and treatment consisted in 5 i.p. injections of 300 μg of Rb4 peptide or vehicle (10% water and 90% PBS), in alternate days, starting the day after tumor cell challenge. It should be noted that in all in vivo experiments, mice showed healthy physical appearance, normal activity levels and normal weight throughout the study period, demonstrating no toxic effects of peptides.

### Rb4 causes necrosis in murine msselanoma cells

**Figure 4:**
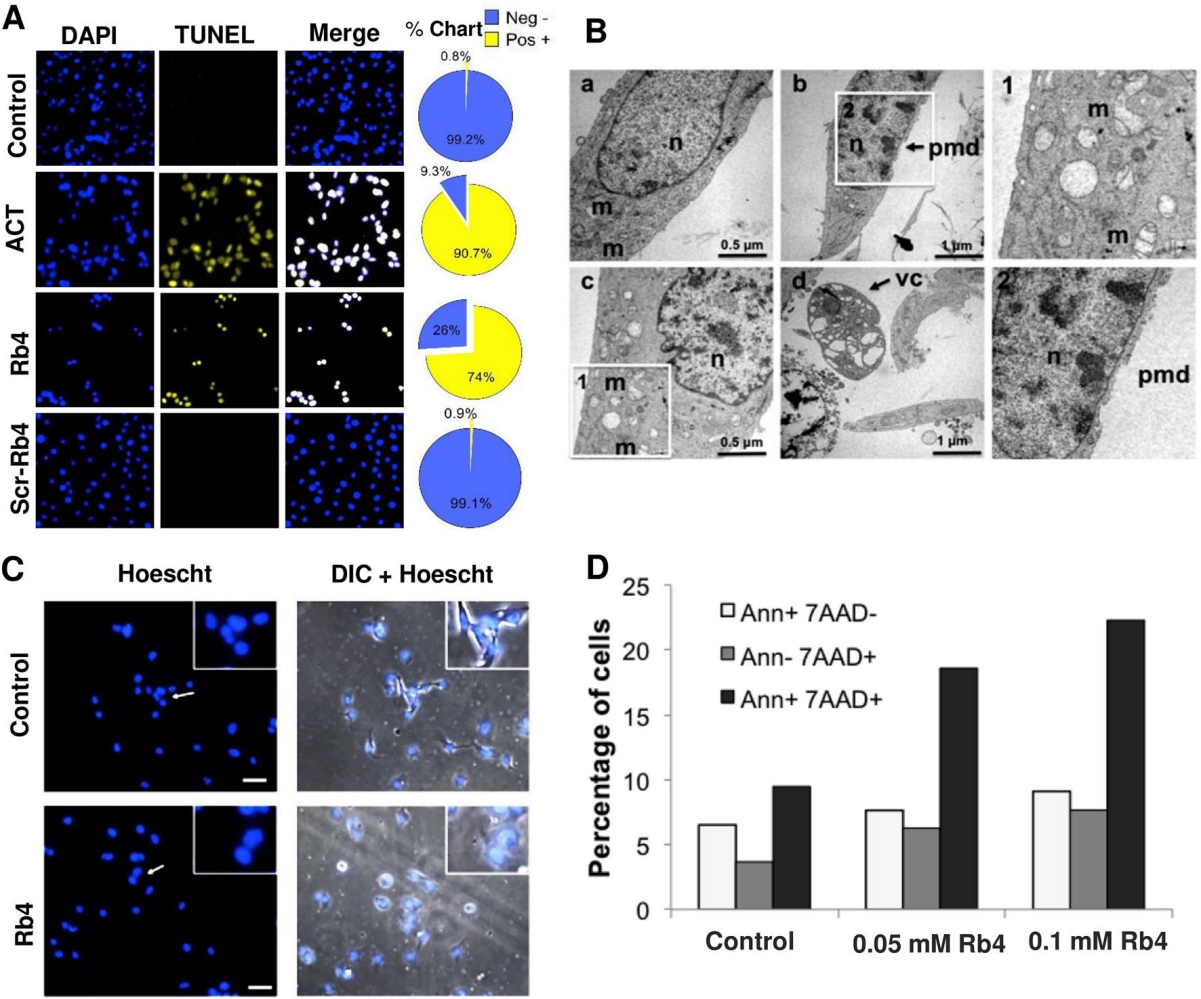
Cell death induced by Rb4 peptide. (**A**) Internucleosomal DNA degradation. B16F10-Nex2 cells were treated with 0.15 mM Rb4 or Scr-Rb4 for 16 h. The DNA of control cells and the treated cells were detected by TUNEL assay in fluorescence microscopy. 1 μg/ml actinomycin-treated (ACT) cells were used as a positive control. Bar 200 μm. Pie charts on the right show the percentage of positive cells (yellow) or negative (blue) of TUNEL assay. (**B**) Ultrastructural characteristics of Rb4-induced cell death. B16F10-Nex2 cells were either left untreated (control, **a**) or treated with 0.15 mM Rb4 for 16 h (**b, c, d, 1, 2**) followed by transmission electron microscopy. Note the presence of dilated mitochondria (**m**), plasma membrane disintegration (**pmd**) and vacuolization of cytoplasm (**vc**) in cells treated with Rb4. (**C**) Hoechst 33342 fluorescent staining to detect apoptotic morphology of B16F10-Nex2 cells after treatment with Rb4 at 0.15 mM for 16 h (arrows indicate cells highlighted in inserts, zoom ×200). Bar, 200 μm. Necrotic cells were recognized by the absence of condensed nuclei. (**D**) Annexin V (Ann) binding in B16F10-Nex2 melanoma cells treated with 0.05 and 0.1 mM Rb4 for 16 h. 7-AAD labeling is also shown. Cells were analyzed by FACS.

Cellular phenotypic alterations induced by Rb4 peptide were observed in B16F10-Nex2 cells treated with 0.15 mM Rb4 but not with the scrambled-Rb4 (Scr-Rb4) peptide. TUNEL assay was used to measure internucleosomal DNA degradation (Fig. 4A). Actinomycin (ACT) treatment, as positive control, stained positive in more than 90% of cells and the Rb4 peptide was also stained positive in approximately 75% of the B16F10-Nex2 cells. Vehicle treatment (PBS) or mock peptide (Scr-Rb4) were inactive, with a positive signal in less than 1% of total cells (Fig. 4A). Along with this experiment, Rb4-treated melanoma cells were also examined by transmission electron microscopy (Fig. 4B). Treated cells exhibited features of necrosis, such as the loss of plasma membrane integrity and absence of nuclear condensation. Transmission electron microscopy did not reveal morphological signs of apoptosis since nuclei appeared largely intact and major chromatin condensation was absent. The vast majority of cells adopted a necrotic morphology with plasma membrane disintegration (Fig. 4B-b,2), swelling of mitochondria (Fig. 4B-c,1) with many hollowed areas, and vacuolated cytoplasm (Fig. 4B-d). The cells were brightly fluorescent, Hoechst-stained nuclei appeared independent of chromatin condensation. Treatment of B16F10-Nex2 cells with 0.15 mM for 16 h led to rounded cells in clusters, with no chromatin condensation (Fig. 4C). In addition, double staining with both Annexin V-PE and 7-AAD or 7-AAD alone identified these necrotic cells (Fig. 4D). Briefly, melanoma cells were treated with 0.05 and 0.1 mM Rb4 during 16 h and stained with 7-AAD and/or Annexin V-PE. While the number of early apoptotic cells (annexin V positive, 7-AAD negative) was not considerably increased with the treatment (0.1 mM Rb4, 9.11%; 0.05 mM Rb4, 7.64%; untreated, 6.52%, white bars), the necrotic cells (annexin V positive, 7-AAD positive, black bars) did increase (0.1 mM Rb4, 22.3%; 0.05 mM Rb4, 18.6%; untreated 9.48%) in a dose-dependent manner. Altogether, these results revealed that Rb4 causes necrosis after 16 h in melanoma tumor cells.

### Rb4 but not Scr-Rb4 triggers PARP-1-mediated necrosis independently of RIP-1 in murine melanoma cells

**Figure 5:**
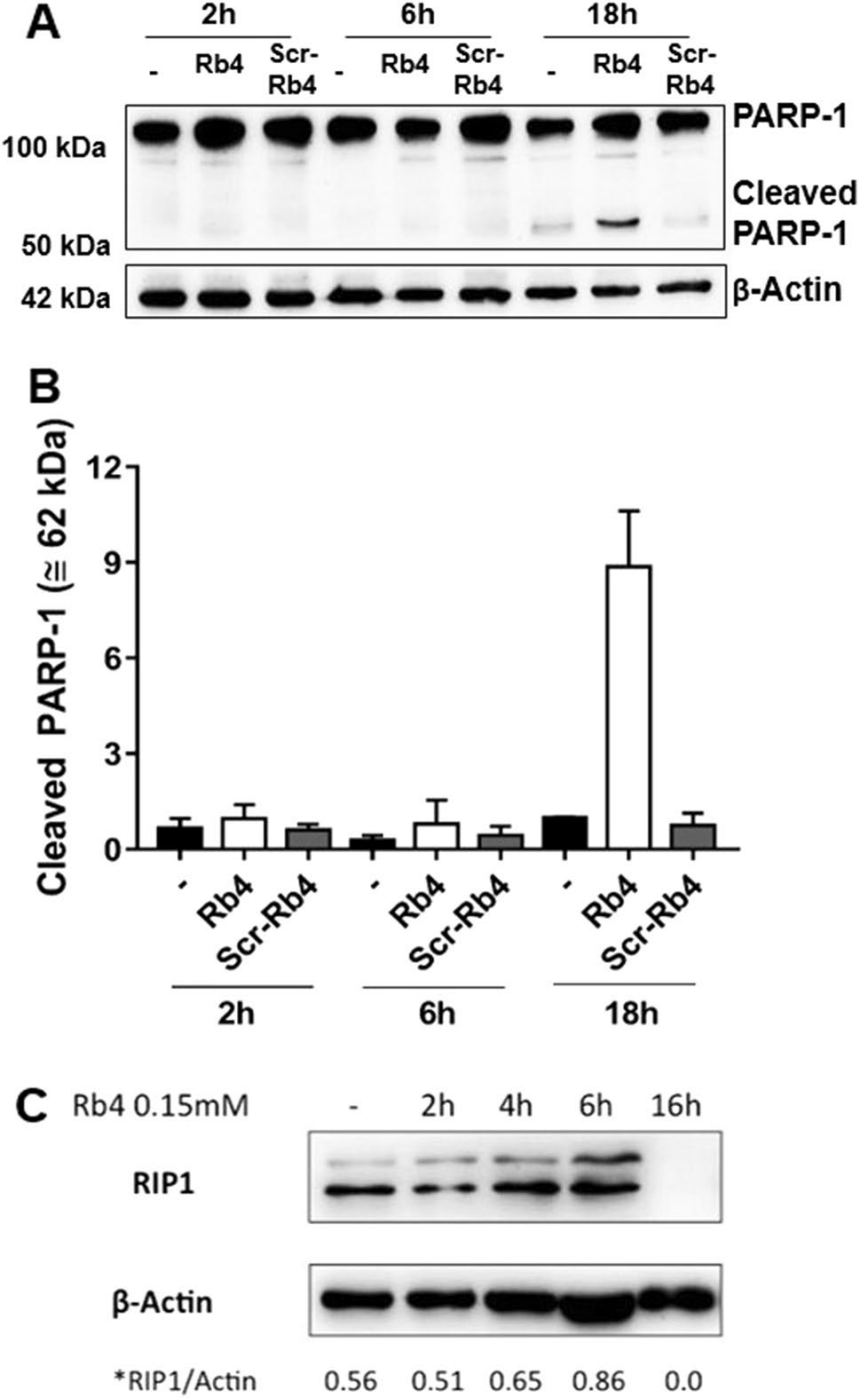
Rb4, but not Scr-Rb4, triggers PARP-1-mediated necrosis independently of RIP-1 in murine melanoma cells. (**A**) Western blotting of PARP-1 after Rb4 treatment of melanoma cells samples analyzed by immunoblotting shows a cleaved band at 62 kDa when compared to Scr-Rb4 and untreated controls. (**B**) PARP-1 cleaved band increased ninefold in Rb4 treated cells when normalized by β–actin expression and compared to both controls. (**C**) Immunoblotting of RIP-1 was also performed showing no RIP1 expression after 16 h. Representative blots from at least three independent experiments are shown. Full length blots are shown in Supplementary Figure S1-online.

**Figure 6:**
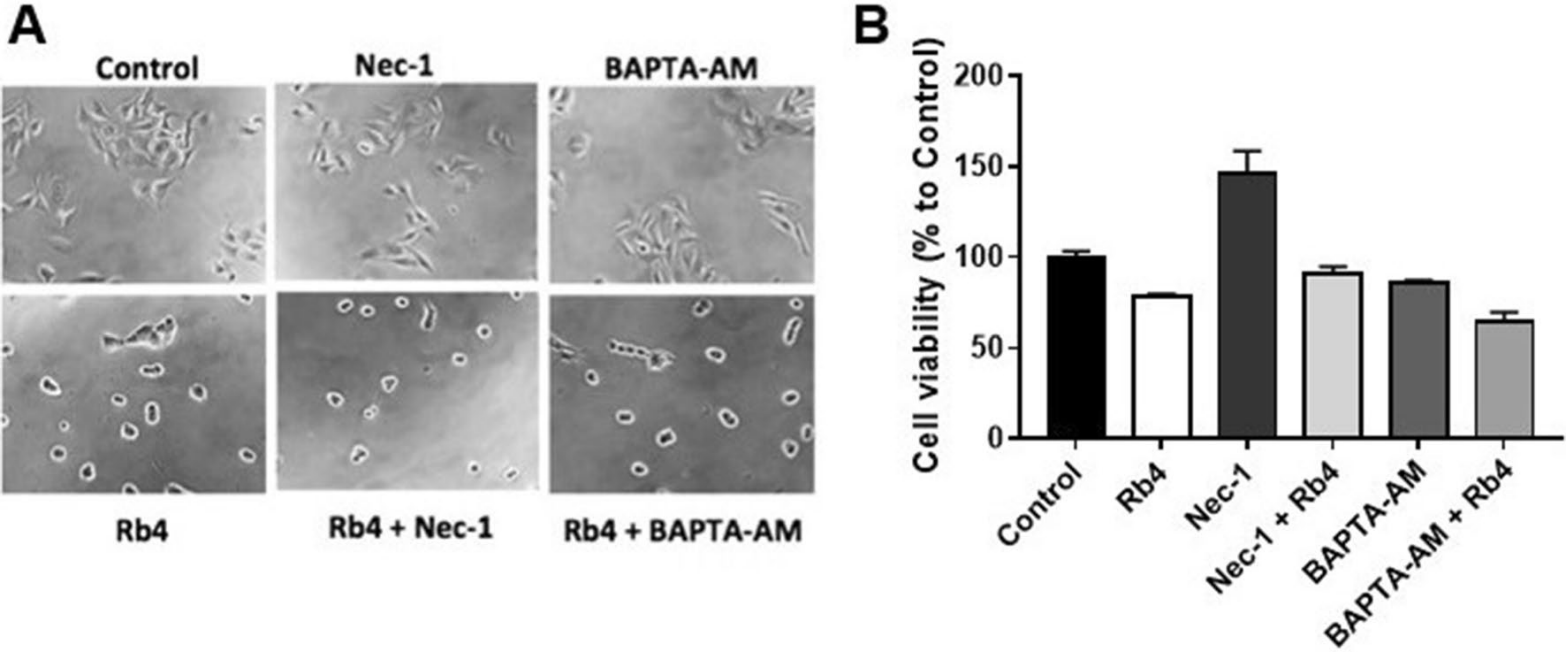
Rb4 peptide effect on the morphology and viability of B16F10-Nex2 cells is independent of the enhanced level of calcium in the cytosol and RIP1 expression. B16F10/Nex2 cells were stimulated with 0.15 mM of Rb4 for 16 h in the presence or absence of 5 µM BAPTA-AM or 100 µM Necrostatin-1 (Nec-1). BAPTA-Am and Nec-1 were added 1 h before Rb4. BAPTA-AM and Nec-1 controls were performed. (**A**) The cell morphologies were determined by interference light microscopy. Images were magnified ×200. (**B**) The cell viability was determined by MTT assay.

A necrotic cleavage of Poly (ADP-ribose) polymerase (PARP-1) has been characterized by Gobeil et al. in Jurkat T cells treated with necrotic inducers such as O_2_, EtOH or HgCl_2_^19^. In our model, melanoma cells showed a degradation band (approximately 62 kDa) of cleaved PARP-1 when incubated with 0.15 mM Rb4 for 16 h and 18 h but not for 2, 4 or 6 h (Fig. 5A,B and supplementary data). No signal of degradation was observed after Scr-Rb4 or PBS treatment for 16 h. The densitometry of degradation bands at 62 kDA showed that Rb4 increased more than ninefold the signal when compared to PBS-mock treatment or Scr-Rb4 peptide incubation (Fig. 5B). In addition, caspase-3 and -9 activity was measured using colorimetric and Western blotting (WB) assays; no caspase activation was detected under the same experimentation conditions (data not shown). Since PARP-1-mediated necrosis might involve RIP1^20^, we also investigated the protein expression by WB. While the expression of RIP1 was little increased at 6 h of incubation with Rb4, cells incubated 16 h with Rb4 surprisingly did not show RIP1 expression (Fig. 5C). These results explain the results obtained with B16F10-Nex2 cells incubated with Rb4, pretreated with 100 µM of necrostatin-1 (Nec-1), a RIP1 activity inhibitor^21^. These cells continued to present the typical morphology of Rb4 treatment, which is clusters of round cells (Fig. 6A). In addition, the cytotoxicity of Rb4 in melanoma cells was not prevented by preincubation with Nec-1 for 1 h (Fig. 6B). These results showed that melanoma cells treated with Rb4 undergo PARP-1-mediated necrosis independently of RIP1.

### Rb4 antagonizes thapsigargin activity on ER calcium flux

The endoplasmic reticulum (ER) plays a key role in maintaining Ca^2+^ homeostasis within the cell^22^. This Ca^2+^ resource is vital for numerous signaling pathways including cell death^23^. The ER has three ubiquitously expressed Ca^2+^ transporters: IP3-R (inositol 1,4,5-trisphosphate-receptor) which releases Ca^2+^ from the ER, RyR (ryanodine receptor) also involved in releasing Ca^2+^ from ER, and SERCA (sarcoplasmic/endoplasmic reticulum Ca^2+^-ATPase) transporter which acquires Ca^2+^ from cytoplasm, transferring it into the ER^24^.

**Figure 7:**
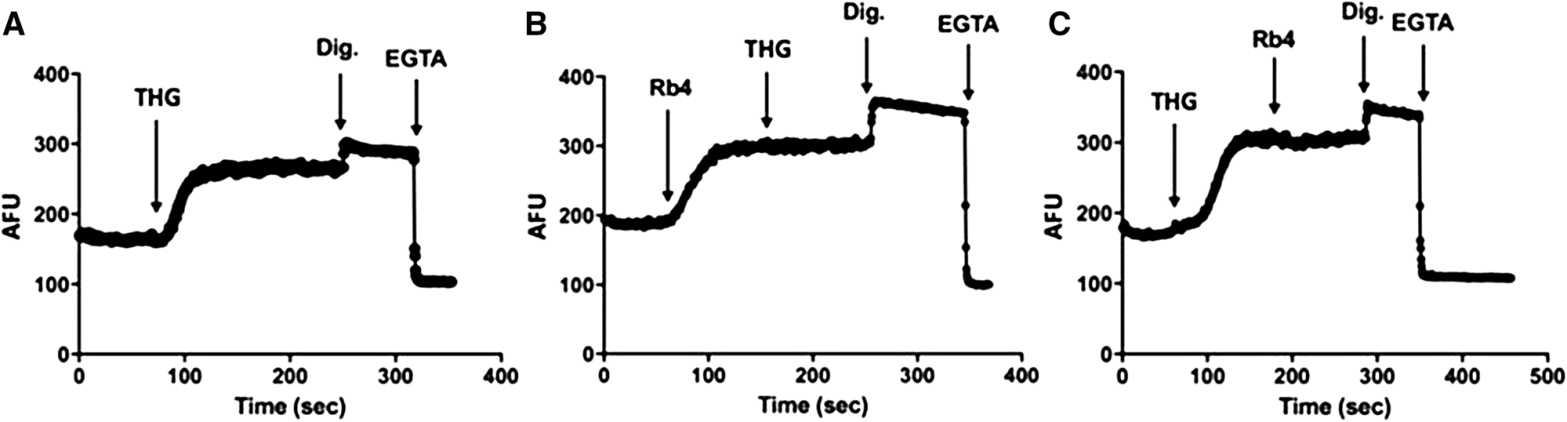
Endoplasmic reticulum calcium flux increased by Rb4. B16F10-Nex2 cells were treated (**A**) with thapsigargin, digitonin and EGTA or (**B**) 0.15 mM Rb4 followed by thapsigargin, digitonin and EGTA or (**C**) with thapsigargin followed by 0.15 mM Rb4, digitonin and EGTA. AFU: Fluo-4 arbitrary fluorescence units. THG: Thapsigargin; Dig: Digitonin.

Rb4 it is a synthetic peptide derived from the protein PLP2, a four-transmembrane domain protein, which has been described to exhibit ion channel characteristics at the endoplasmic reticulum^15,16^. We hypothesized that Rb4 treatment, added as a single peptide, might interfere in the calcium flux to the ER. We, therefore, examined the peptide effect on the cytosolic Ca^2+^ in B16F10-Nex2 cells (Fig. 7). By using the fluo-4AM dye, we monitored the cytosolic Ca^2+^ levels in Ca^2+^-free medium after thapsigargin (THG) addition (Fig. 7A), thus reflecting the kinetics of Ca^2+^ release after sarcoplasmic/endoplasmic reticulum Ca^2+^-ATPase (SERCA) inhibition^25^. In addition, B16F10-Nex2 cells were incubated with 0.15 mM Rb4 and pre-incubated or post-incubated with THG. Immediately after the incubation of Rb4, the Ca^2+^ peak increased and the cytosolic Ca^2+^ did not change in presence of THG (Fig. 7B). The same was observed when Rb4 was incubated after THG (Fig. 7C). These results suggest that the ER supplies the calcium and that Rb4 could function as a competitor of THG activity in ER calcium flux (inhibitor of SERCA transporter activity) resulting in the blocking of cytoplasmic Ca^2+^ uptake into ER. Altogether, the results suggest that Rb4 increases calcium in the cytoplasm of murine melanoma cells, which originates from ER.

The importance of calcium for Rb4-cytotoxicity was also evaluated in B16F10-Nex2 cells pretreated for 1 h with 5 μM BAPTA-AM^26^ (an intracellular calcium chelator) before Rb4 incubation. B16F10-Nex2 cells treated with 0.15 mM for 24 h, preincubated with BAPTA-AM showed the same cellular morphological alterations caused by Rb4 (Fig. 6A), and the decrease of Rb4-treated melanoma cells viability was not prevented by the calcium chelator (Fig. 6B). This result demonstrates that calcium does not play a central role in Rb4-cytotoxicity in B16F10-Nex2 cells.

### Rb4 affects actin dynamics in murine melanoma cells

The synthetic peptide Rb4 significantly reduced B16F10-Nex2 melanoma growth by triggering PARP-1-mediated tumor-cell necrosis. Furthermore, Rb4-treated melanoma cells show increased cytosolic Ca^2+^ released by the ER. Actin dynamics have been shown to regulate Ca^2+^ released from the ER^27^. Furthermore, actin depolymerization and polymerization was described to regulate RIP1-independent necrotic death on U87MG cells expressing Bcl-xL elicited by a non-selective isopeptidase inhibitor^28^.

**Figure 8:**
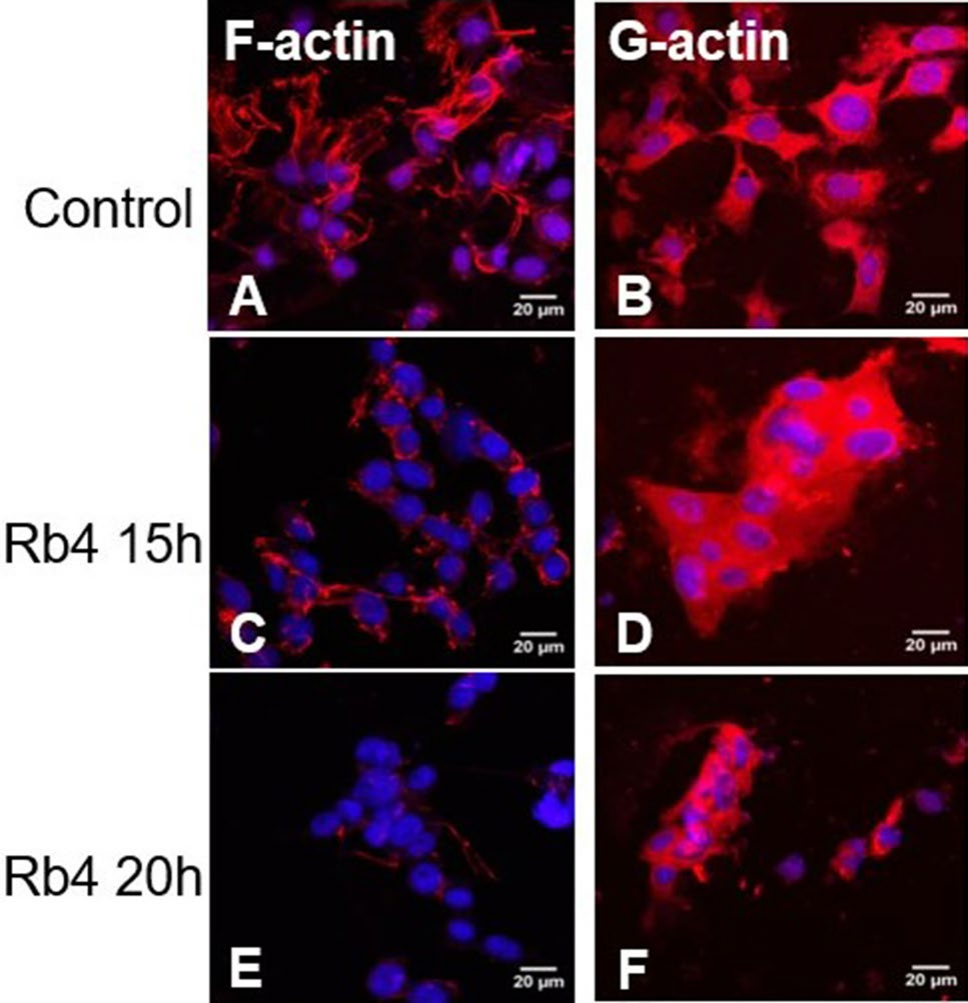
Rb4 affects actin polymerization and depolymerization. B16F10-Nex2 cells were treated with 0.15 mM Rb4 for 15 h or 20 h. After incubation, the cells were fixed, permeabilized and incubated with phalloidin-rhodamine and DAPI (**A**,**C**,**E**) to stain filamentous actin. Note the close to full loss of F-actin structure in C and E after Rb4 treatment for 15 and 20 h. B16F10-Nex2 cells were also fixed, permeabilized and incubated with Dnase I (**B**,**D**,**F**) to stain globular actin. Images are representative fields from coverslips analyzed by confocal microscopy detection.

To address whether Rb4 influences actin polymerization, melanoma cells were evaluated by confocal microscopy after 15 h and 20 h of 0.15 mM Rb4 treatment with F-actin and G-actin staining. The complete loss of morphology was shown to be accompanied by depolymerization of almost all F-actin and the accumulation of G-actin on treated cells (Fig. 8) suggesting that Rb4 significantly alters actin dynamics, polymerization and the F-actin stabilization.

### Rb4 induces the production of DAMPs

Dying and stressed cells secrete, release or undergo surface expression of DAMPs. Particular DAMPs serve as powerful immunological adjuvants and mediate immunogenic cell death (ICD)^29,30^. The exposure and release of calrecticulin^31^ and HMGB1^32^ has been described on pro-apoptotic, post-apoptotic and/or necrotic cells.

**Figure 9:**
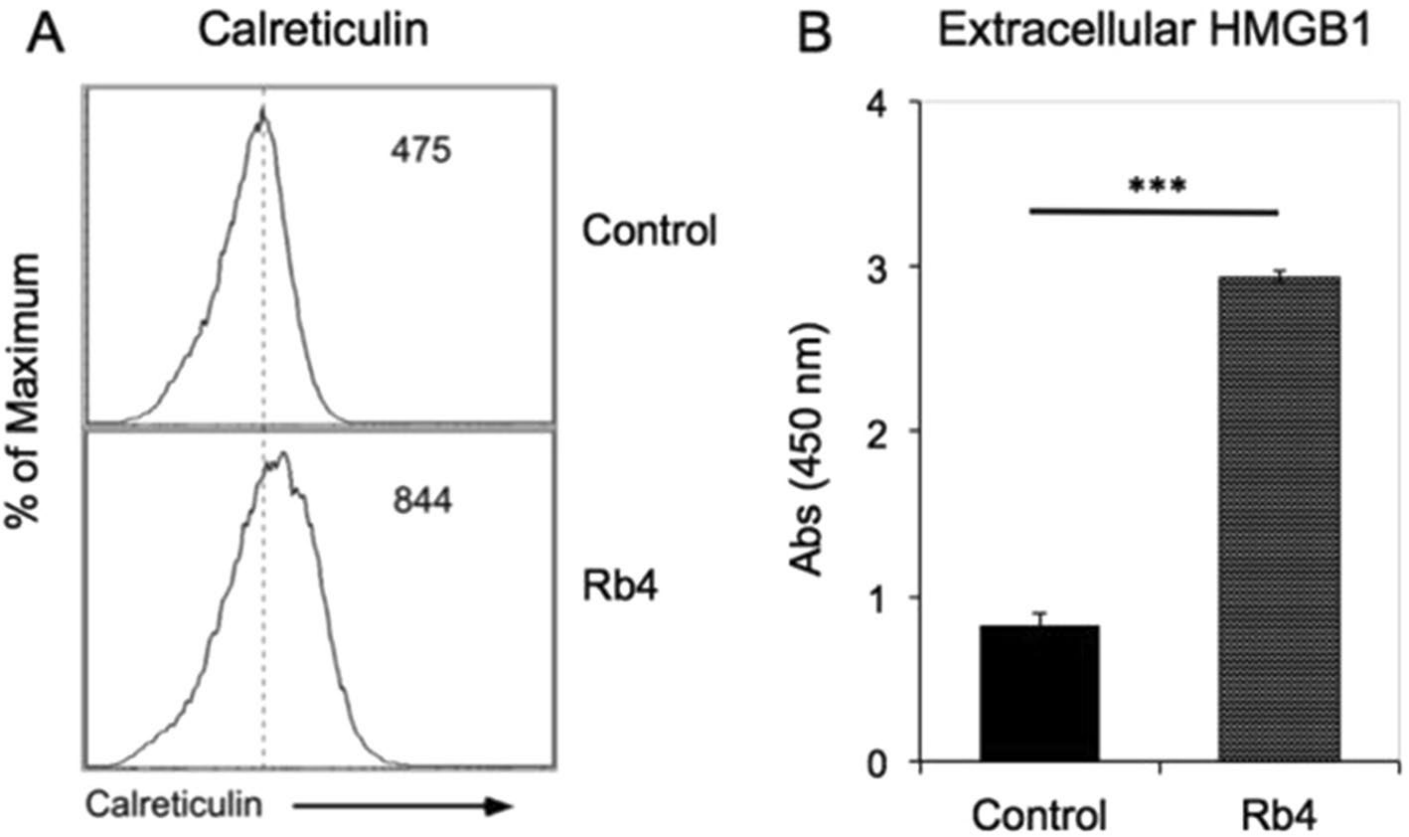
Expression of DAMPs by Rb4 treatment. B16F10-Nex2 cells were treated with 0.15 mM Rb4 for 16 h. (**A**) Calreticulin expression of control cells and treated cells was detected by flow cytometry. (**B**) The HMGB1 expressions in control and treated cells were detected by ELISA assay. Two-tailed t-test was performed, ***p < 0.001.

In our model, calreticulin surface expression was assessed by flow cytometry (Fig. 9A). B16F10-Nex2 cells treated with Rb4 showed 1.77-fold increase of calreticulin on the cell surface as compared to non-treated cells. In parallel, we also examined the effects of the peptide on the HMGB1 release and surface exposure of calreticulin. With this purpose, B16F10-Nex2 cells were treated with 0.15 mM Rb4 for 16 h. ELISA was used to measure HMGB1 released into the medium (Fig. 9B). The results showed that HMGB1 significantly (p < 0.001) increased 3.54-fold in the medium of Rb4-treated B16F10-Nex2 cells compared with non-treated cells. The data shows Rb4 treatment could also promote increase in the expression of two DAMPs.

## Discussion

Peptides are being increasingly explored as antitumor agents due to various characteristics. They have several advantages over some other therapeutic agents such as easy availability by synthesis, convenient purification and storage, low molecular weight, efficient immunoreactivity and the ability to specifically target tumor cells with decreased toxicity^1^. Peptides have been reported to inhibit angiogenesis^33^, to have the ability to target metastases^34^ and to elicit cytotoxic T-lymphocyte responses^6^.

In this work we investigate a PLP2 N-terminal-derived peptide named Rb4. PLP2, a four-transmembrane domain protein, has an important role in tumor formation and metastasis of murine B16F10 melanoma in a syngeneic model^17,18^. We have compared the cytotoxicity of the peptide in both tumorigenic and nontumorigenic cells. In vitro*,* Rb4 exhibits cytotoxic activity against some cancer cell lines, especially against the murine melanoma B16F10-Nex2 cell line. No activity was observed for the scrambled Rb4 peptide (Scr-Rb4) in all the performed tests. In addition, toxicity of the peptide was analyzed after 24 h in non-tumorigenic cells in culture. The murine non-tumorigenic cell line MEF was unaffected by the highest concentration of Rb4 (1 mM). Our group has determined in a protein microarray assay that the B16F10-Nex2 cell line over-expresses PLP2^35^, which could justify the cytotoxic effect of Rb4 peptide in this cell lineage by competing with the endogenous protein. On the other side, the expression of PLP2 on A2058 human melanoma cells could be down-regulated, decreasing the sensitivity of these cells to Rb4. Ding et al.^36^ demonstrated that miRNA (miR-664) downregulated PLP2 expression in human melanoma cells by directly targeting the PLP2 untranslated region, mediating ​direct suppression of PLP2 expression. Further, the importance of PLP2 in the invasion and tumorigenicity of B16F10 cells has been shown by others^17,18^.

The mechanism of Rb4 cytotoxicity in murine melanoma cells has been studied in the present work. Here, we show that the peptide induces necrosis in melanoma cells as confirmed by dilated mitochondria, plasma membrane disintegration, and vacuolization of cytoplasm, DNA fragmentation, absence of chromatin condensation, increase of double staining with Annexin V/7-AAD but not with Annexin V only, and absence of caspase-3 and -9 activation (data not shown). Rb4-treated murine melanoma cells showed necrotic cleavage of PARP-1, as characterized by Gobeil et al.^19^ in Jurkat T cells treated with necrotic cell inducers. Since PARP-1-mediated necrosis might involve RIP1^20^, we looked for RIP1 expression and found that until 6 h of incubation with Rb4, RIP1 increased slightly, while with 16 h RIP1 expression was not detected, suggesting that the cell death pathway triggered by Rb4 leads to inhibition of RIP1 over time. In addition, neither the morphological alteration nor the viability of Rb4-treated B16F10-Nex2 melanoma cells was inhibited by Nec-1. These results suggest that PARP1-mediated necrotic death of B16F10-Nex2 cells triggered by Rb4 is independent of RIP1 expression. Luan et al.^37^ have shown that RIP1 plays a critical role in survival of human melanoma cells undergoing pharmacological ER stress induced by thapsigargin (THG). While RIP1 is upregulated in melanoma cells relatively resistant to THG-induced apoptosis, knockdown of RIP1 rendered cells susceptible to be killed by THG^37^. Our results suggest that Rb4 leads to necrotic cell death of murine melanoma cells mainly through the induction of ER stress and RIP1 inhibition.

Rb4 is a synthetic peptide derived from the protein PLP2, a four-transmembrane domain ion transporter protein localized in the endoplasmic reticulum^15,16^. Rb4 increases the cytosolic calcium from the ER as demonstrated in Fig. 6. On one hand, both Rb4 and THG alone increased cytosolic calcium levels considerably. Incubation with each one, however, did not alter the levels of calcium increased by the other. These results suggest that Rb4 might act as an inhibitor of SERCA activity. The cytosolic calcium increase is not central for the Rb4-cytotoxicity in melanoma cells since neither the morphological alteration nor the viability of Rb4-treated B16F10-Nex2 cells was inhibited by BAPTA-AM, an intracellular calcium chelator.

Actin dynamics has been shown to regulate Ca^2+^ release from endoplasmic reticulum^27^ and is associated with tumor necrosis factor-induced apoptosis^38^. Furthermore, actin depolymerization and polymerization was described to regulate RIP1-independent necrotic death on U87MG cells expressing Bcl-xL elicited by a non-selective isopeptidase inhibitor^28^. The treatment with Rb4 leads tumor cells to a morphology loss characterized by F-actin depolymerization and G-actin monomers accumulation. These results clearly suggest that the peptide interferes in the G-actin pool affecting the conversion to filamentous, the stability of F-actin on tumor cells and overall actin dynamics. Similarly, leukemia HL-60 cells were already shown to respond to UV irradiation with early and transient actin polymerization and, later, with actin depolymerization on F-actin pools which were shown to be essential elements in the formation and release of apoptotic bodies^39^.

The Rb4 peptide when peritoneally injected significantly reduced the number of lung metastatic nodules in the syngeneic B16F10-Nex2 melanoma model. The Rb4 also reduced the volume of subcutaneously grafted B16F10-Nex2 melanoma tumor in syngeneic mice. Moreover, the survival rate of mice treated with Rb4 was significantly prolonged in comparison to that of the vehicle control groups (Fig. 3B). In vivo activity of Rb4 in murine melanoma depends on the immune system since its protective activity against metastatic melanoma B16F10-Nex2 observed in immunocompetent mice was not observed in immunocompromised (NOD-SCID-IL-2R-*gamma null*) mice. The expression and release of DAMPs in Rb4-treated melanoma cells could explain the absence of activity of Rb4 in immunocompromised mice. Rb4-treated cells showed 3.54-fold secreted HMGB1 in relation to untreated cells and 1.77-fold increase of cell surface calreticulin. Once present in the extracellular microenvironment, DAMPs function as “danger signals” in the immune system. Calreticulin is a chaperone multifunctional protein, predominantly found in the lumen of the ER, and associated with several physiological and pathological processes in cells. The main functions include chaperone activity and regulation of Ca^2+^ homeostasis. As a result, calreticulin could be involved in cellular Ca^2+^ uptake into the ER via SERCA, Ca^2+^ storage within the ER, and Ca^2+^ release from the ER^24^. On the other side, cell surface calreticulin ensures the phagocytic removal of dying cancer cells by a subset of DCs, being a pre-requisite for the development of adaptive anticancer immunity. The release of high-mobility group protein B1 (HMGB1) activates DCs via the inflammasome or Toll-like receptor pathways^40^. The screening for anticancer drugs and treatments in cancer cells revealed that their ability to induce immunogenic cell death (ICD) depends on the induction of ER stress^31^.

The present work provides evidence that Rb4, derived from PLP2, triggers necrotic cell death in murine melanoma cells, in a dose-dependent manner, through ER stress caused by inhibition of SERCA transporter activity, which leads to alteration of actin dynamics and RIP1 inhibition. Hence, the capacity of Rb4 to lyse tumor cells in a non-physiological (unconventional) fashion may contribute to its pro-immune effects through the liberation of DAMPs such as HMGB1 and calreticulin. These DAMPs would provoke local inflammation and immune reactions in the tissues resulting in a therapeutic effect against metastatic and subcutaneous murine melanoma.

Since many anticancer drugs signal through mitochondria, ER stress is a potential target for the development of drugs^41,42^. Therefore, we suggest that Rb4 peptide is a promising anticancer agent for the treatment of drug-resistant tumors.

## Material and methods

### Ethics statement

The Ethical committee of Federal University of São Paulo (UNIFESP), CEP no. 1234/2011 approved all experimental protocols with mice in accordance with the relevant international guidelines (Fapesp Project 2010/51423-0). All the experiments were carried out in compliance with the ARRIVE guidelines.

### Peritumoral treatment of subcutaneously grafted murine melanoma and of experimental lung metastasis

Six-week-old male C57BL/6 mice obtained from the Center for Development of Experimental Models (CEDEME), Federal University of São Paulo (UNIFESP) (average weight of 25–28 g), were subcutaneously injected with 5 × 10^4^ B16F10-Nex2 tumor cells. Peritumoral injections were given starting 24 h after tumor cell graft. Treated groups (5 animals per group) received daily doses of 300 μg of Rb4 and the control group received PBS. Tumor size was measured daily for 2 weeks with a caliper, using the formula V = 0.52 × D1^2^ × D2, where D1 and D2 are the short and long tumor diameters respectively. The maximum allowed tumor volume was 3000 mm^3^, before euthanasia. In the *Experimental Lung Metastasis* model, 6-week-old male C57BL/6 mice or NOD/Scid-IL2-Rγ^null^ mice were challenged intravenously with 5 × 10^5^ syngeneic B16F10-Nex2 melanoma cells/mouse (0.1 ml). For protection experiments five groups of five animals received on days 1, 3, 5, 7, 9 after tumor cell challenge, intraperitoneal doses of 300 μg of Rb4 (approximately 10 mg/kg/mice) and the control group received the peptide vehicle. After 20 days, lungs were collected from animals of each group and inspected for metastatic colonization.

### Cell lines and culture

The murine melanoma cell line B16F10 was originally obtained from the Ludwig Institute for Cancer Research (LICR, São Paulo, Brazil) and the subline B16F10-Nex2 was isolated at the Experimental Oncology Unit (UNONEX), Federal University of São Paulo, UNIFESP, deposited at Banco de Células do Rio de Janeiro (BCRJ-0342). Human melanoma (A2058), colon carcinoma (HCT-8) and breast carcinoma (MCF-7) cell lines were provided by the LICR, São Paulo, Brazil. Human cervical carcinoma cell line (HeLa) and human glioblastoma cell line (U87-MG) were provided by Hugo P. Monteiro, UNIFESP, and Osvaldo K. Okamoto, University of São Paulo, respectively. Murine syngeneic, colorectal adenocarcinoma (CT26) and murine pancreatic (PANC) cells were provided by Dr. Guillermo Mazzolini from the School of Medicine of Austral University, Buenos Aires, Argentina. The mouse embryonic fibroblasts (MEF) and NIH-3T3 were gifts from Luis F. Lima Reis, Hospital Sirio-Libanes, São Paulo. Tumor cells were cultured at 37 °C in a humidified atmosphere containing 5% CO_2_, in RPMI 1640 medium (Invitrogen, Carlsbad, CA) supplemented with 10 mM N-2-hydroxyethylpiperazine-N′-2-ethanesulfonic acid (Hepes) (Sigma, St. Louis, MO), 24 mM sodium bicarbonate (Sigma), 40 mg/l gentamicin (Schering-Plough, São Paulo, Brazil). HeLa, U87-MG, CT26, NIH-3T3 and MEF cells were maintained in DMEM supplemented as for the RPMI-1640 medium. All cell lines were previously checked for mycoplasma using Lonza’s MycoAlertTM Mycoplasma Detection Kit (Catalog #: LT07-118). All cell lines were free from the presence of contaminants prior to use.

### Peptides

Peptide Rb4 (MADSERLSAPGCWAACTNFSRTRK) and Scr-Rb4 (LACTNCRTSDAMWEKFSRPSAGRA) were purchased from Peptide 2.0 (Chantilly, VA). A stock solution of 1 mM was prepared by diluting the peptides, amidated at the C terminus, in RPMI 1640 medium with 10% distilled water.

### Time-lapse live cell microscopy

B16F10-Nex2 cells (3 × 10^5^) were plated in RPMI 1640 medium on 35 mm glass bottom dishes and kept for 5 h at 37 °C in a humidified atmosphere containing 5% CO_2_. A stock solution of Rb4 and Scr-Rb4 peptides were diluted to 0.15 mM in RPMI 1640 and used to replace the medium in the cell plates. B16F10-Nex cells were transferred to a Nikon Biostation IMQ equipped with a 20× objective (NA 0.8) and a high-sensitivity camera for imaging of large bright fields-of-view. Cells were followed for up to 72 h when kept at 37 °C in a CO_2_ incubation chamber on the equipment. Data acquisition started up to 1 h after placing the specimens into the Biostation IMQ, after system stabilization at 37 °C and 5% CO_2_. Time-lapse phase images were taken every 20 min for 24 to 72 h with five fields for each condition. Images captured were made into a movie using the BioStation IM version 2.12 software at a rate of approximately 10 frames per second and processed using Nikon Elements software. Movies depicted on Supplementary Material represent composed images of 24 h elapsed time.

### Cytotoxicity assay in vitro

Rb4 was diluted in supplemented RPMI medium with 0.5% dimethyl sulfoxide (DMSO, SIGMA) and incubated with 5 × 10^3^ or 1 × 10^4^ murine and human tumor cells in 96-well plates. After a pre-incubation period (16 h), cell viability was assessed using the Cell Proliferation Kit I (MTT) (Boehringer Mannheim), a 3-(4,5-dimethylthiazol-2-yl)-2,5-diphenyltetrazolium bromide-based colorimetric assay. Readings were made in a multiplate reader (SpectraMax) at 570 nm. Necrostatin-1 (Nec-1; methyl-thiohydantoin-tryptophan (MTH-Trp)) was added 1 h before the peptide incubation, in some cytotoxicity assays.

### Transmission electron microscopy

B16F10-Nex2 cells (5 × 10^4^) were cultivated on plastic disks made from Aclar film. Cells were then incubated with 0.15 mM Rb4 for 16 h at 37 °C and fixed in a solution of 2.5% glutaraldehyde and 2% formaldehyde in 0.1 M sodium cacodylate buffer, pH 7.2, at room temperature for 20 h. Cells were then washed in the same buffer for 10 min, fixed with 1% osmium tetroxide in 0.1 M cacodylate at pH 7.2 for 30 min, and washed with water for 10 min at room temperature. Subsequently, cells were treated with an aqueous solution of 0.4% uranyl acetate for 30 min and washed with water for 10 min. After fixation, cells were dehydrated in graded ethanol (70, 90, and 100%), treated quickly with propylene oxide, and embedded in EPON. Semi-thin sections from selected regions were collected on grids and stained in alcoholic 1% uranyl acetate and in lead citrate prior to examination in a Jeol 100 CX electron microscope (Tokyo, Japan).

### Chemiluminescence-ELISA

A 96-well opaque plate (Nunc, Roskilde, Denmark) was coated with supernatant (0.2 ml) of 0.15 mM Rb4-treated B16F10-Nex2 cells, overnight (16 h), at 4 °C. The plate was washed with 0.05% Tween 20-PBS (T-PBS) and blocked for 4 h at RT with 1% BSA. Anti-HMGB1 antibody (Abcam), at 1:500, was then added overnight. After incubation, the plate was washed extensively with T-PBS, and the secondary HRP 1:500 anti-Rabbit IgG (Invitrogen, Thermo Fisher Scientific) was added and incubated for 2 h. The plate was washed 5 times with T-PBS, and the reaction was evaluated by Absorbance using OPD (Sigma Aldrich) in a multiplate reader (SpectraMax) at 450 nm.

### Chromatin condensation

Tumor cells (10^4^), cultivated overnight on round glass coverslips, were treated with 0.15 mM Rb4 for 16 h, washed with PBS, and fixed for 30 min at room temperature with 2% formaldehyde. The cells were washed with PBS and stained with 2 µM Hoechst 33342 (Invitrogen) for 10 min. Cells were visualized in a Biostation IM-Q (Nikon) fluorescence microscope at 60× magnification. Images were processed with ImageJ. Apoptotic cells are characterized by the presence of chromatin condensation and DNA leakage into the cytoplasm of tumor cells.

### TUNEL assay

TUNEL (TdT-mediated dUTP-X nick end labeling) was performed according to the manufacturer's instructions (In Situ Cell Death Detection kit, Fluorescein; Roche Applied Science). Briefly, 1 × 10^4^ cells were cultivated in round glass coverslips and treated for 2 h with 0.15 mM Rb4 or Scr-Rb4 peptides. Cells were fixed in 2% formaldehyde for 30 min at room temperature. They were then permeabilized with 0.1% Triton X-100 for 30 min at room temperature and incubated with terminal deoxynucleotidyl transferase (TdT) in the reaction buffer with dUTP-fluorescein at 37 °C for 1 h, then stained with 10 μg/ml DAPI for 10 min and visualized in an Olympus BX-51 fluorescence microscope with immersion oil, at 60× magnification. Images were processed with ImageJ.

### Phosphatidylserine translocation

Tumor cells (5 × 10^5^) were cultivated in 6-well plates with 0.05 and 0.1 mM Rb4 peptide for 16 h at 37 °C and 5% CO_2_. Treated and untreated cells (1 × 10^6^) were harvested with trypsin and were incubated with binding buffer (10 mM HEPES/NaOH, pH 7.5, 140 mM NaCl, and 2.5 mM CaCl_2_) in the presence of 7-AAD and annexin V (Annexin V-PE Apoptosis Detection kit; Sigma) for 10 min at room temperature and analyzed by flow cytometry (Becton–Dickinson FACSCanto II apparatus) with FACSDiva software.

### Cytosolic Ca^2+^ determinations in spectrofluorimeter

B16F10-Nex2 cells (1 × 10^6^), washed twice with HBSS buffer supplied with 1.3 mM CaCl_2_, were incubated for 60 min at 37 °C in the same buffer and 5 µM of the calcium indicator Fluo-4 AM (Molecular Probes) and 1 mM of probenecid (Sigma), which minimizes indicator extrusion and compartmentalization. Subsequently, the cells were washed twice with the same buffer, without CaCl_2_, and transferred to a quartz cuvette. Intracellular calcium was determined using a Hitachi F-7000 spectrofluorimeter (Tokyo, Japan) by continuous measurement of the fluorescence variation at λex = 505 nm and λem = 530 nm. The intracellular calcium increased with the addition of 10 µM thapsigargin (THG). Rb4 peptide at 0.15 mM was added before and after THG. Maximal fluorescence (Fmax) was determined after the lysis of cells with 33.3 µM of digitonin (Sigma), and minimal fluorescence (Fmin) was determined after adding 3 M EGTA in Tris, pH 8,7 until no further decrease in fluorescence was observed. AFU = arbitrary fluorescence units.

### Confocal microscopy for actin polymerization and depolymerization

B16F10-Nex2 cells (1 × 10^4^) were plated on round glass coverslips and incubated overnight previous to incubation with 0.15 mM of Rb4 peptide for 15 and 20 h. After treatment, cells were washed three times with PBS, fixed with 3.7% paraformaldehyde for at least 30 min and permeabilized in 0.1% Triton X-100 for 30 min followed by blocking for 1 h with 150 mM NaCl, 50 mM Tris, and 0.25% BSA (Sigma-Aldrich). Cells were stained with phalloidin-rhodamine (1:1000) (Invitrogen) for 1 h or with deoxyribonuclease I-Alexa Fluor 594 conjugate (27 µg/ml) (Invitrogen) for 1 h followed by 10 µg/ml DAPI (Invitrogen) for nucleic acid staining for 10 min. The coverslips were mounted on slides with Vectashield (Sigma) and analysed in a Confocal Leica SP5 microscope, with a 63 × 1.4 oil objective; the Z series was obtained according to sampling criteria built in the software. DAPI was examined at 350-nm excitation and 470-nm emission and the phalloidin-rhodamine, for filamentous actin (F-actin) staining, or deoxyribonuclease I-Alexa Fluor, for globular actin (G-actin) staining, was examined at the excitation/emission at 540/565 nm and at 590/617.

### Tumor cell lysate

B16-Nex2 melanoma cells untreated and treated with the peptide were harvested and resuspended in PBS (5 × 106 cells) with protease inhibitors. RIPA Lysis Buffer (Sigma-Aldrich) was used for cell disruption. Light microscopy and Trypan blue exclusion staining verified the method’s efficiency. The cell lysate was kept at − 80 °C for later use.

### Western blotting analysis

Western blottings were run with proteins from total cell lysates (40 μg). They were separated by 10% SDS–polyacrylamide gel electrophoresis and transferred to Immobilon P transfer membrane (Millipore, Darmstadt, Germany). The membranes were washed in Tris-buffered saline with Tween (10 mM Tris–HCl, pH 8, 150 mM NaCl, and 0.05% Tween 20) and blocked overnight at 4 °C with 5% nonfat milk in Tris-buffered saline with Tween 20. The blots were probed overnight at 4 °C with mAbs from Cell Signaling, Boston, MA; Bioss-bs336BR Woburn, MA; Santa Cruz, Dallas, TX; ABCAM, Cambridge, UK; as indicated. After 2 h incubation with horseradish peroxidase-conjugated secondary antibody, immunoreactive proteins were detected by enhanced chemiluminescence (ECL; Amersham Biosciences, Little Chalfont, UK). Bands densitometry was obtained using ImageJ software. Protein concentrations were determined by Bradford assay (Bio-Rad, Hercules, CA). Supplementary Figure S1 online shows the full-length blots.

### Statistical analysis

Statistical significance was examined by the unpaired two-tailed Student’s *t*-test. The Log-rank (Mantel–Cox) test was used to analyse long-term survival curves. The *P*-value < 0.05 was considered as statistically significant.

## Supplementary Information

Supplementary Figure 1.

Supplementary Video 1.

Supplementary Video 2.

Supplementary Video 3.

## Data Availability

The raw data supporting the conclusions of this article will be made available by the authors, without undue reservation, to any qualified researcher.
