# Supplementary material for: PLP2-derived peptide Rb4 triggers PARP-1-mediated necrotic death in murine melanoma cells
Source: Sci Rep. 2022 Feb 21;12:2890. doi: 10.1038/s41598-022-06429-8 (PMC8861012; doi:10.1038/s41598-022-06429-8)
Supplement: Supplementary file 1 — Supplementary Figure 1. [file 41598_2022_6429_MOESM1_ESM.docx]

**PLP2-derived peptide Rb4 triggers PARP-1-mediated necrotic death in murine melanoma cells**

Vera S. C. Maia^1^, Rodrigo Berzaghi^2^, Denise C. Arruda^3^, Fabrício C. Machado^1,2^, Leticia L. Loureiro^2^, Pollyana M. S. Melo^4^, Alice S. Morais^1*^, Alexandre Budu^4^, Luiz R. Travassos^1,2#^

*^1^Recepta Biopharma São Paulo, Brazil*

*^2^Experimental Oncology Unit, Department of Microbiology, Immunology and Parasitology, Federal University of São Paulo, São Paulo, Brazil*

*^3^Integrated Group of Biotechnology, University of Mogi das Cruzes, UMC, Mogi das Cruzes, SP, Brazil*

*^4^Department of Biophysics, Federal University of São Paulo, São Paulo, Brazil*

^#^ *In memoriam* to Prof. Luiz R. Travassos, M.D, PhD

^*^Correspondence author to:

Alice S. Morais

alice.morais@receptabio.com.br

Supplementary information

Supplementary Figures and Legend


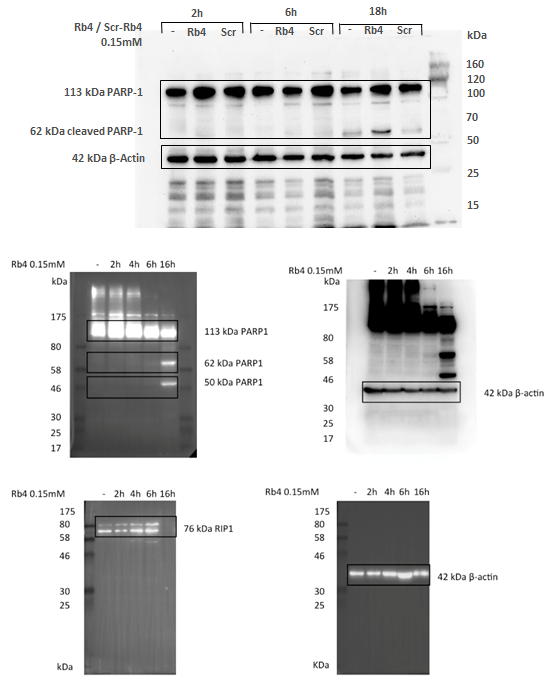


**Supplementary Figure S1. Full-length blot images for PARP-1 (above) and RIP-1 (below) used for Figure 5.** In the above blot, PARP-1, cleaved PARP-1 and β-actin were detected within the same blotting. In the two middle panels, high exposition and proteins amounts shows PARP-1 (molecular weight: 113 kDa), cleaved PARP-1 with two bands (62 kDa and 50 kDa) and β-actin (molecular weight: 42 kDa) detected, within the same blotting in the right panel. In the last two blots, RIP-1 (molecular weight: 76 kDa) and β-actin (molecular weight: 42 kDa) were detected with the gels/blots processed in parallel and the same amount of protein used. Different blots with different samples derived from similar experiments were also performed. The black lined rectangles delineate the cropped bands shown in the main Figure 5.
